# Cancer in the news: Bias and quality in media reporting of cancer research

**DOI:** 10.1371/journal.pone.0242133

**Published:** 2020-11-09

**Authors:** Amanda Amberg, Darren N. Saunders

**Affiliations:** School of Medical Sciences, University of New South Wales, Sydney, NSW, Australia; National Cancer Institute, UNITED STATES

## Abstract

Cancer research in the news is often associated with sensationalised and inaccurate reporting, which may give rise to false hopes and expectations. The role of study selection for cancer-related news stories is an important but less commonly acknowledged issue, as the outcomes of primary research are generally less reliable than those of meta-analyses and systematic reviews. Few studies have investigated the quality of research that makes the news and no previous analyses of the proportions of primary and secondary research in the news have been found in the literature. We analysed distribution of study types, research sources, reporting quality, gender bias, and national bias in online news reports by four major news outlets in USA, UK and Australia over six-months. We measured significant variation in reporting quality and observed biases in many aspects of cancer research reporting, including the types of study selected for coverage, the spectrum of cancer types, gender of scientists, and geographical source of research represented. We discuss the implications of these findings for guiding accurate, contextual reporting of cancer research, which is critical in helping the public understand complex science, appreciate the outcomes of publicly-funded research, maintain trust, and assist informed decision-making. The striking gender bias observed may compromise high-quality coverage of research by limiting diversity of opinion, reinforces stereotypes and skews public visibility and recognition towards male scientists. Our findings provide useful guidelines for scientists and journalists alike to consider in providing the most informative and accurate reporting of research.

## Introduction

Cancer is complex and challenging to study, and news reporting on cancer research is susceptible to hype, contradiction and misinformation. Clearly communicating the outcomes and context of research is key to helping non-specialists understand complex science, and assisting patients and families make informed decisions about modifying risk and treatment selection. Poor reporting practice may have serious consequences for public and scientific communities alike, including the generation of false or unmet expectations, potentially fuelling disappointment and a loss of trust in science [1, 2]. Few studies have focused on quantifying the types and quality of scientific research that gain attention in the news, which is arguably as important as accurate translation of research paper to news story. A preference towards reporting novel primary research stories with low replication likelihood often result in the refutation (or failed replication) of a research finding getting significantly less attention than the initial finding itself [3], reinforcing an ‘asymmetry of bullshit’ [4].

Primary research more easily lends itself to ‘breakthrough’ headlines since, by definition, it presents original data. Quality and reliability are not intrinsic features of meta-analyses and systematic reviews but depend on appropriate systematic methods [5, 6]. Nevertheless, the nature and purpose of these forms of secondary research–collating, comparing and re-analysing a set of primary studies to reduce uncertainty–render them less susceptible to error than individual primary studies [7]. Based on this assumption, secondary research is an important source of science news for the general public, yet there are indications of a reporting bias favouring primary studies [8]. In academic publishing, peer review and the accumulation of primary research papers followed by meta-analyses and review articles are designed to help filter inaccuracies of individual study outcomes but there is no equivalent formalised system in the news media beyond standard editorial oversight.

It may be tempting for researchers, journalists, philanthropic bodies and research institutions to sensationalise scientific findings in their pursuit of funding, readers or publicity and a common consequence of poor quality reporting is a hype cycle characterised by false expectations and subsequent disappointment [1]. Hype may be generated by journalists, institutional press releases, or the scientists themselves, and can then be amplified through the media cycle [9, 10]. As far as we know, this is the first combined analysis of study type distribution, reporting quality, and other biases in cancer research reporting.

## Methods

### News report collection

Twenty news reports were collected from each of the online versions of *The Guardian* (UK edition), The *New York Times* (*NY Times*), *The Sydney Morning Herald* (*SMH*) and the *Australian Broadcasting Corporation* (*ABC*), beginning in March 2017 (with the latest published in September 2017), generating a total dataset of 80 reports. The following search terms were used within the search function on each source’s website: *‘cancer study’*, *‘cancer research’*, *‘cancer science’*, *‘targeted therapy’*, *‘cancer screening’*, *‘cancer screening study’*, *‘tumour research’*, *‘tumor research’*, *‘cancer treatment’*, *‘cancer genetics’* and *‘cancer scientists’*. Only original reports were included in the sample, reports re-published from other news sources were excluded to avoid overlap in the data. General reports which dealt with cancer-related topics but which did not base the discussion on a specific study were excluded. When several studies were discussed and no one central study could be distinguished, the report was excluded. No other inclusion/exclusion criteria, filters or qualifiers were applied. Details of individual reports (including Pubmed ID (PMID), quality scores and hyperlinks to reports) are contained in S1 Table.

### Classification and scoring

The study discussed in each media report was traced and classified as basic research, clinical research, epidemiological research, meta-analysis or systematic review according to the classification of Röhrig *et al*. [11]. News report quality was scored according to a matrix based on eleven criteria adapted from previous studies (Table 1) [10, 12]. Australian cancer incidence and mortality statistics were obtained from the Australian Institute of Health and Welfare [13] N.B. relative rates in US and UK are very similar). DALY data were obtained from the Global Burden of Disease Study (2015) (Institute for Health Metrics and Evaluation http://ghdx.healthdata.org/record/ihme-data/gbd-2015-cancer-incidence-mortality-ylls-ylds-dalys-1990-2015). Gender of senior authors (i.e. names appearing in first and last position on author list) and quoted experts in each report was also quantified using pronouns quoted in reports or on homepage. Source nationality was classified according to primary academic affiliation of corresponding authors.

**Table 1:** News report quality scoring criteria.

Criterion	Scoring	Notes
Peer reviewed research source	0, 3 (no, yes)	Peer review was assigned a heavier weighting than other binary criteria to reflect importance.
Conflicts of interest or funding source identified	0, 1 (no, yes)	Conflicts of interest or funding sources had to be mentioned in the news report to meet this criterion.
Independent expert(s) quoted	0, 1 (no, yes)	Independent experts must not be affiliated with the paper, publishing journal, research institute or funding body.
Link to research source	0, 1 (no, yes)	Links must lead directly to the research source (published paper, conference abstract, et cetera). Links to journal homepage received 0 for this criterion.
Traceable research source	0, 1 (no, yes)	Enough information provided to allow tracing the source within 5 min.
Study limitations identified	0, 1 (no, yes)	Required mentioning a limitation of the study’s method, evidence, conclusion or implications. General statements about what the study did not aim to investigate were not sufficient to fulfil this criterion.
Placed study in broader research context	0, 1 (no, yes)	The report should refer to related knowledge or theories generated by researchers unaffiliated with the study in focus.
Absolute risks or benefits quantified	0, 1 (no, yes)	Risks and benefits presented by a study should be described in absolute numbers. Percentages or total incidence did not fulfil this requirement. This criterion was not applicable to some reports. For these, the total scores were adjusted as a proportion of the maximum: (assigned score÷16)×17.
Misleading headline	0, 1 (yes, no)	This included sensationalising, incorrect or otherwise misleading headlines.
Emphasis maintained	0–3	The main aims, outcomes and implications as presented in the study should be maintained in the headline and body of the news report. The scoring range was as follows: emphasis maintained in neither headline nor body (0), in either headline or most of body (1), in both headline and most of body (2), in both headline and all of body (3).
Avoided overgeneralisation	0–3	Overgeneralisation could refer to sample populations, the targets of a treatment or other aspects of the study depending on its classification and topic. Scores were allocated as follows: both headline and body overgeneralising (0), either headline or body overgeneralising (1), headline and body mostly avoided overgeneralisation (2), headline and all of body avoided overgeneralisation (3).

### Data analysis

Chi-square or Fisher’s exact tests were used to compare the categorical variables. Quality scores were aggregated according to Table 1 and differences in mean scores was tested using one-way ANOVA, for multiple comparisons with a Tukey test. When analysing national bias, all studies that had been conducted as international collaborations were included in the ‘international’ category.

## Results

### Content bias

**Fig 1:**
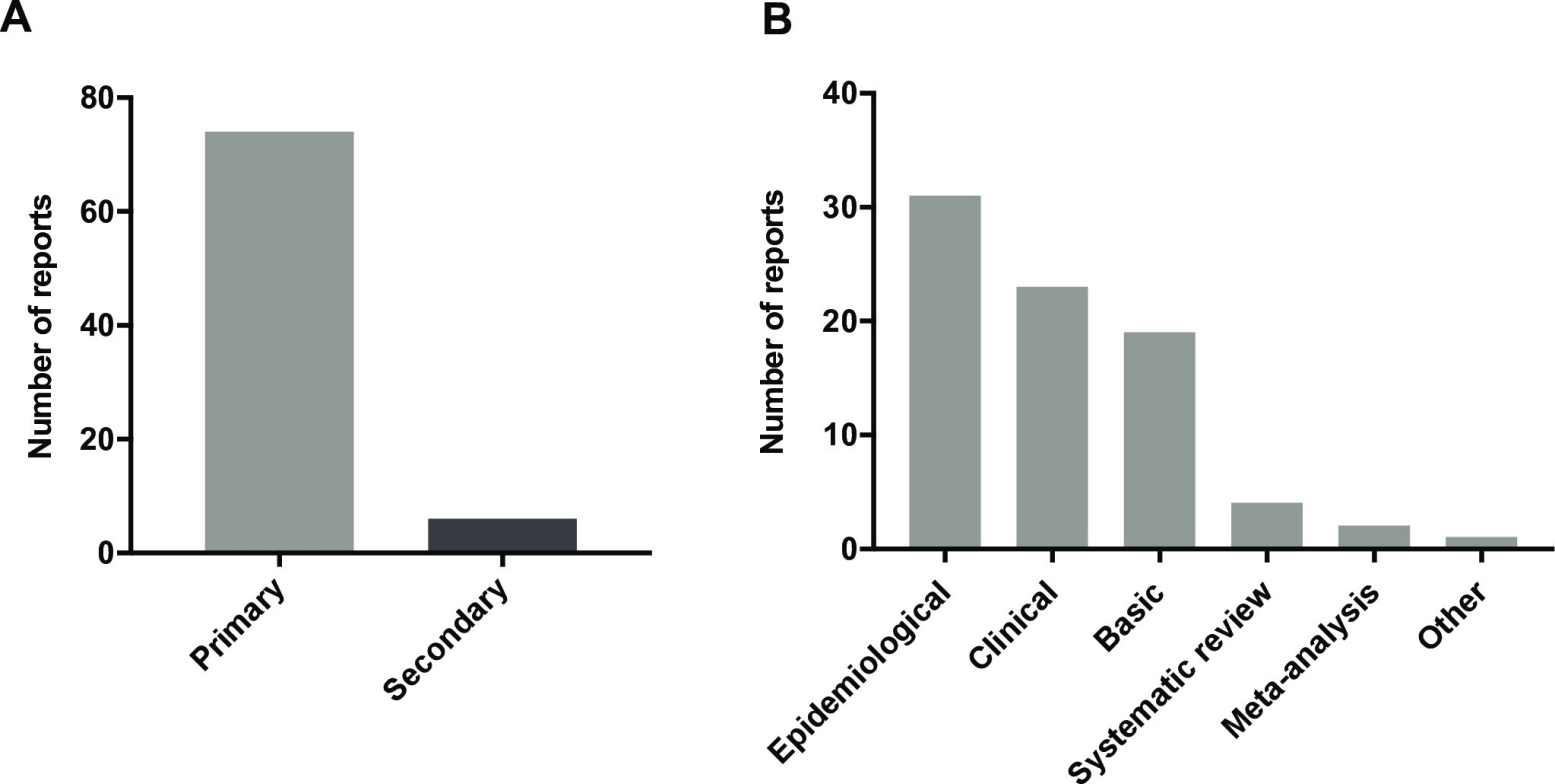
Study type bias represented in online news reports about cancer: **A.** Frequency of primary and secondary research represented in online news reports about cancer research. **B.** Distribution of research subtypes represented in news reports (n = 80).

The long-term implications of primary research findings are rarely known at the time of publication. A remarkable proportion of published results will be refuted by further investigation or subsequent meta-analyses will report notably smaller effect sizes [7, 14–16]. Similar trends have been observed in basic medical research, where only a small fraction of the most encouraging early findings end up in clinical use [17]. Quality and style have been shown to vary across news outlets [18, 19], but even the largest newspapers with the best overall standards tend to cover more studies with poorer methodology and observational studies over RCTs or systematic reviews [20–22]. In our cohort, 92.5% of reports (74/80) were based on primary research studies (Fig 1A). When studies were further classified by subtype, epidemiological studies were the most prevalent overall, accounting for 38.75% of reports (31/80), followed by clinical and basic research at 28.75% (23/80) and 23.75% (19/80) respectively (Fig 1B). Secondary studies consisted of four systematic reviews and two meta-analyses (Fig 1B). One study did not fit in any of the categories and was therefore classified as ‘other’.

### Cancer types represented in reporting

**Fig 2:**
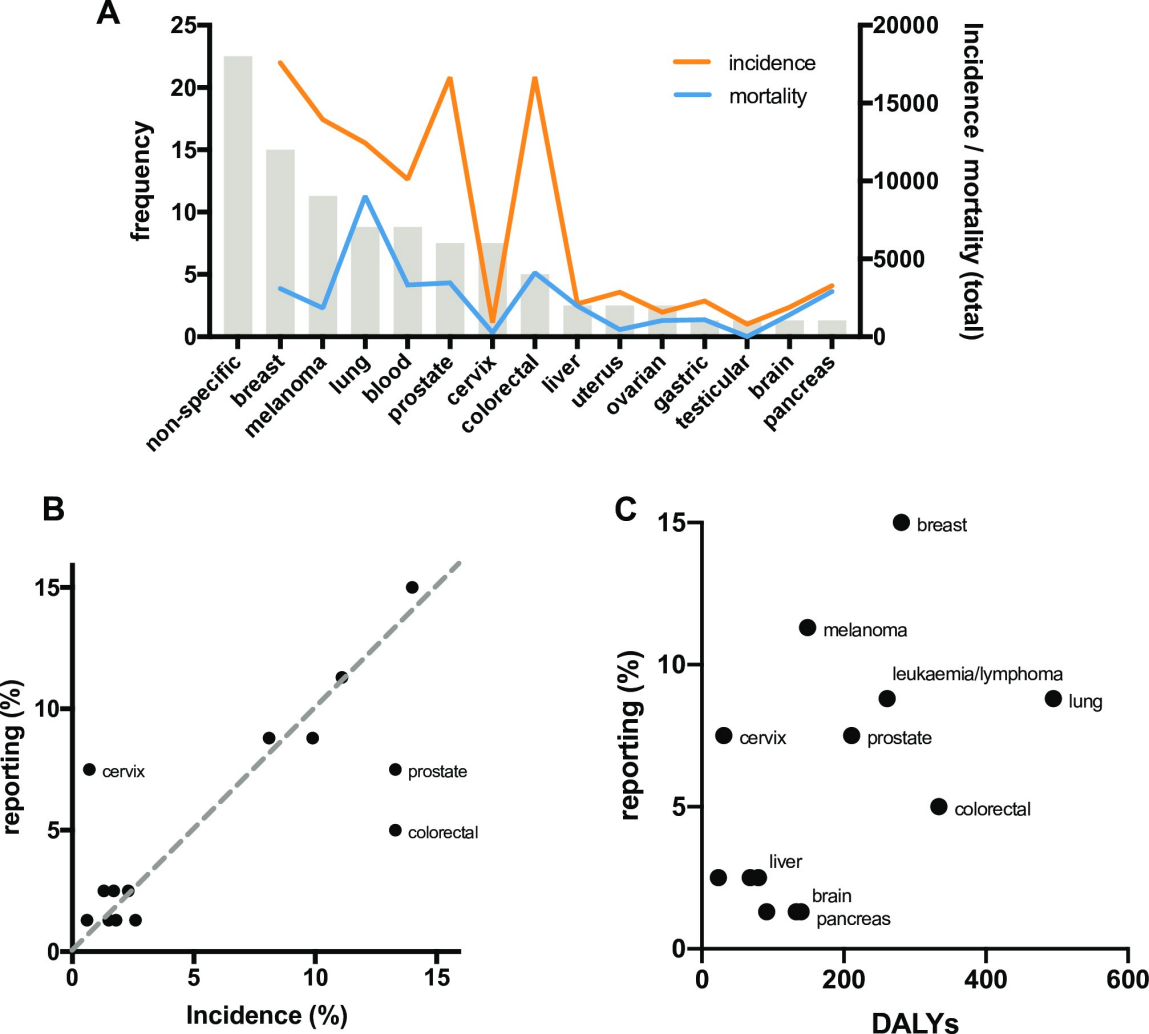
Analysis of bias in cancer types reported. (**A)** Distribution of cancer types in research studies covered in news reports, with Australian incidence and mortality rates; Reporting frequency (as a proportion of total) compared with relative incidence **(B)** and mortality **(C)**.

We examined the distribution of cancer types (defined by anatomical site) represented in our cohort of 80 news reports. The most frequent category observed was non-specific (i.e. not related to a specific cancer type), representing 18/80 (22.5%) reports and possibly reflecting a strong bias towards more basic research on disease mechanisms and risk. Among cancer types explicitly identified in news reports, breast (15%), melanoma (11.3%), lung (8.8%) and blood (8.8%) cancers were the most frequently reported (Fig 2A). Reports specifically mentioning gastric, testicular, brain and pancreas cancer were the least frequently observed, with each only being represented in a single report. Many cancer types were not represented in news reports at all during sample period. We observed a strong correlation between reporting and incidence of specific cancer types (R^2^ = 0.594, p = 0.0013) but not with mortality (Fig 2B and 2C). Research on cervical cancer was reported more frequently than would be expected relative to incidence, while prostate and colorectal cancer were under-represented in news reports (Fig 2B). Relative to mortality, cervical, melanoma and breast cancer were over-represented, while lung, pancreas, and colorectal cancer were under-represented (Fig 2C).

### Reporting quality

**Fig 3:**
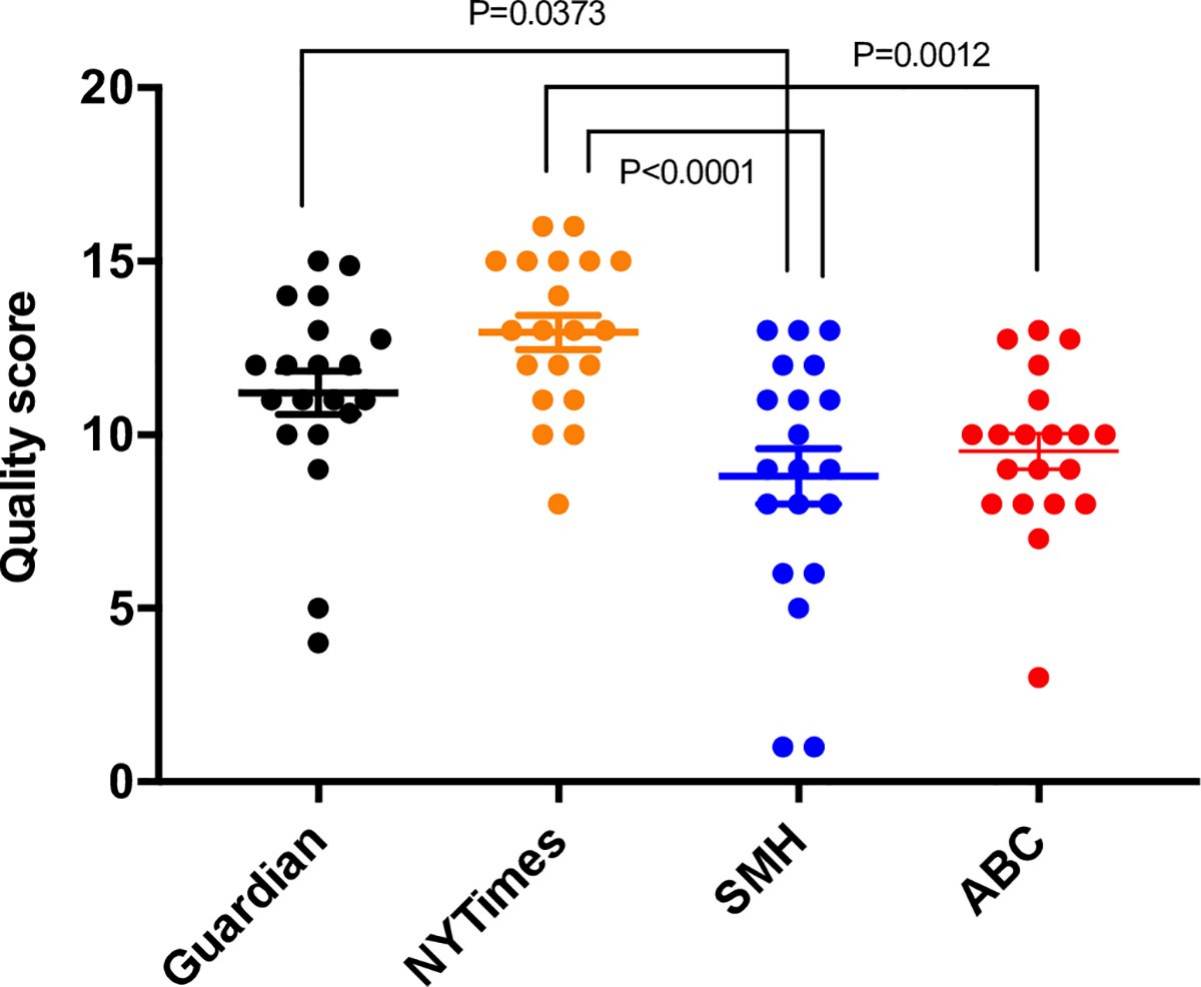
Quality scores of news reports on cancer research. Each point represents the score of an individual news report. Bars indicate the mean ± SEM for each news outlet (n = 20 for each).

News reporting of research is of most value to readers if it accurately conveys the outcomes, context and implications of the research. Accurate reporting is critical for informing decisions on modifying risk, choice of intervention, understanding prognosis, etc. The level of consensus between news articles and related original research papers as a marker of accuracy has been the focus of extensive research [12, 23–27]. Today’s online environment is thought to result in many major news outlets utilising the same sources of information, potentially resulting in an amplified spread of poor quality reporting [28]. Research institutes and funding bodies seeking publicity and philanthropic support may exploit this space as press releases play a major role in shaping the content of many news articles [10]. We measured reporting quality in our cohort using a scoring matrix modified from Singer [12] and Taylor [10] (Table 1). The *NY Times* had the highest average quality score (12.9), while the lowest average scores were seen in the Australian news sources (9.5 and 8.8 for *ABC* and *SMH* respectively), with reports in *The Guardian* averaging a quality score of 11.2 (Fig 3).

Limitations of research were often omitted from news reports across the cohort, but this problem was more apparent in reports about basic research, with 16/19 reports not discussing limitations of the research being reported. Funding sources or conflicts of interest were not identified in any of the news reports on clinical research. Coverage of studies that were not peer reviewed was a relatively common feature of reporting on clinical trials (7/17 studies) and epidemiological research (12/22 studies).

### National bias in reporting of cancer research

While research performed in the USA dominates scientific output in terms of papers published [29], reporting on research in a local context has important implications for both consumers and scientists alike. Different risk factors may have proportionally different significance for various audiences and it is critical that scientists are able to reach the most relevant audiences on topics of local importance. For example, understanding the role of UV exposure in melanoma risk is an important consideration in Australia. Further, it is important for both scientists and consumers alike to have outcomes of publicly funded research communicated to taxpayers.

**Fig 4:**
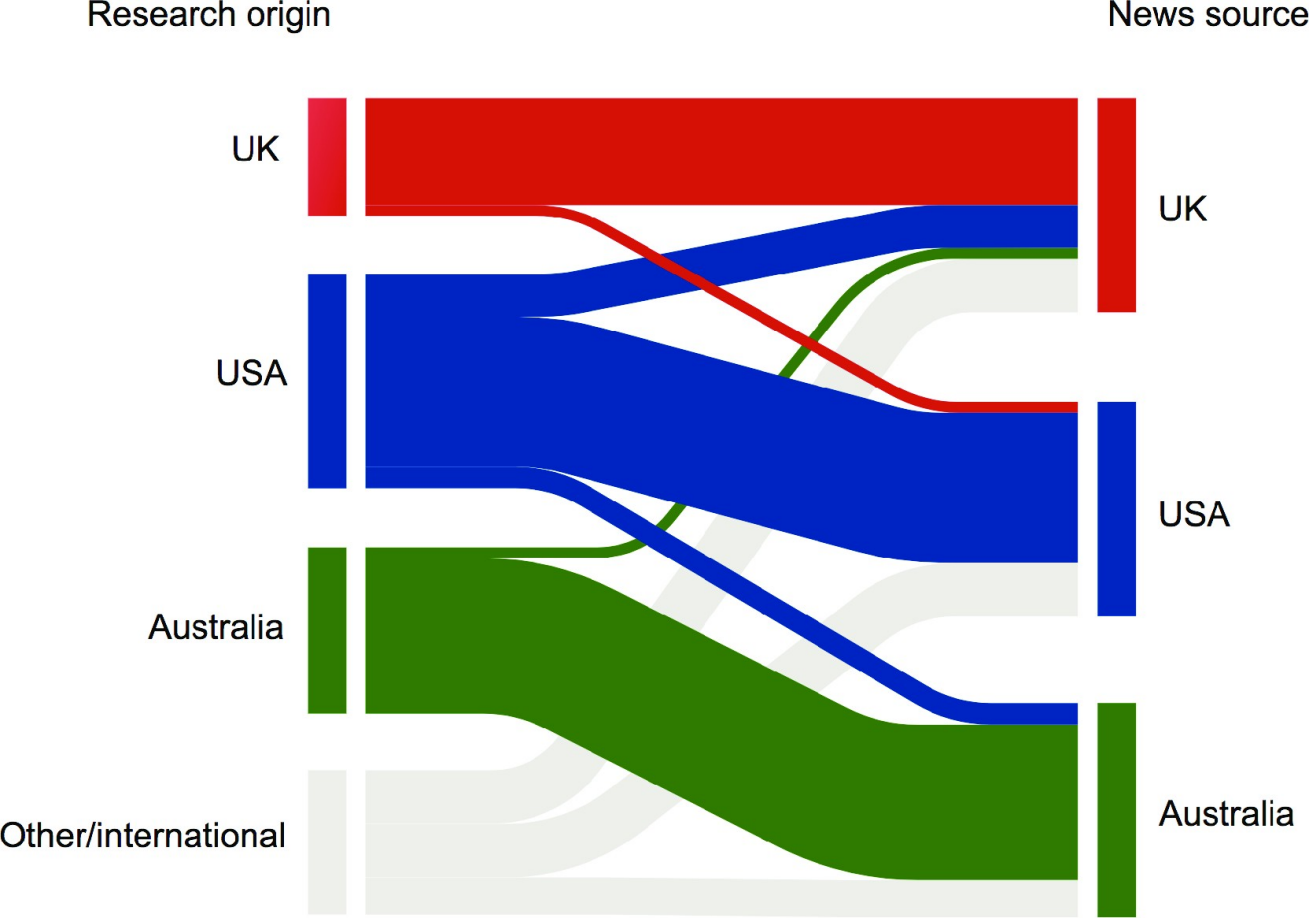
National bias in reporting of cancer research. Sankey chart showing relationships between country of research origin (left) to country in which reporting news organisation is based (right), with bar sizes representing proportional representation. The UK is represented by *The Guardian*, USA by *NY Times* and Australia by *SMH* and *ABC*. Designation of research source as “other/international” refers to studies where the primary authors were based in countries other than the UK, Australia or USA.

To analyse national bias in cancer research reporting, we analysed the country of origin of research cited in each news report—determined by the primary affiliation of the corresponding author of the research publication (where available). At least half of the reports in each news source were based on research from the same country in which the news organisation was based (Fig 4). *The Guardian* (UK edition) had the most diverse national origin of research cited, with only 50% of reports based on research performed primarily in the UK. In contrast, 70% of reports in the *NY Times* were based on research studies performed primarily in the US and 72.5% of Australian news reports were based on Australian research (65% and 80% by *SMH* and *ABC*, respectively) (Fig 4). Viewed from the opposite perspective, the great majority (29/30 studies, or 97%) of Australian research studies represented in our cohort were only reported in Australian news outlets. In other words, only 1/30 Australian studies received international coverage (Fig 4).

### Gender bias in reporting of cancer research

**Fig 5:**
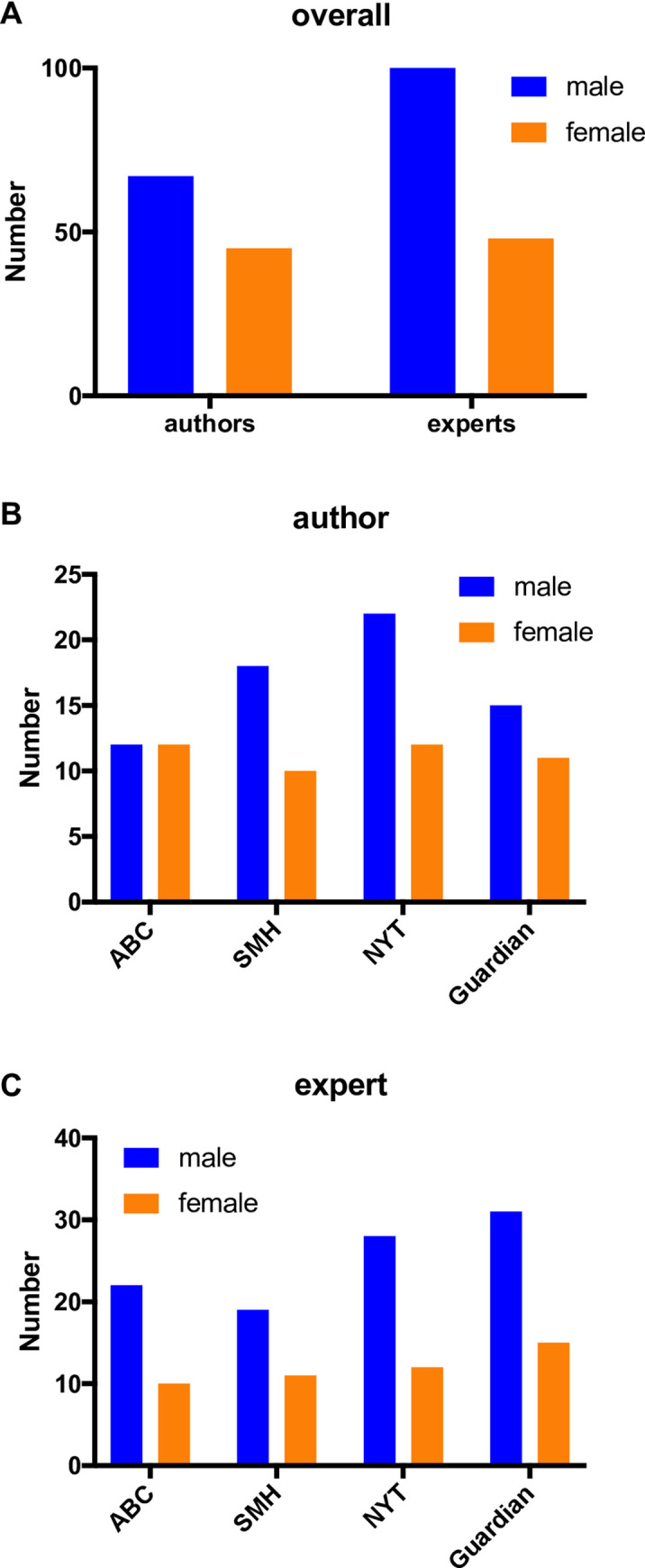
Gender bias in senior authors and quoted experts in news reports. **(A)** Distribution of senior authors and experts across the entire cohort (n = 80), and distribution of authors **(B)** and experts **(C)** in individual news outlets (n = 20 each).

Female scientists face a suite of documented biases [30–33], and a number of studies have established that women are under-represented in news media [34, 35]. More specifically, the systematic under-recognition of female scientists–known as the *Matilda effect* [36]–has been demonstrated in science communication, where publications from male authors are associated with greater perceived scientific quality [37]. Across our entire cohort of 80 news reports, we observed a significant gender bias among senior authors, with 60% (67/112) of research studies reported having male senior authors (Fig 5). We also observed a significant bias toward male experts being quoted in news reports, with 68% (100/148) of quoted experts being male. A similar trend was observed in individual news outlets, with the exception of the *ABC*—where equal representation of male and female senior authors was observed in the studies forming the basis of news reports (Fig 5). The bias towards quoting male experts in online reports about cancer research was consistent across individual news outlets (Fig 5).

## Discussion

Clear communication of research outcomes in context is important in helping non-specialists understand the often complex and challenging contents of scientific publications. Poor reporting may hinder informed decision-making about modifiable risks and treatment selection, generate false or unmet expectations and undermine trust in science [1, 2]. When searching for novel stories, journalists are likely to favour primary research findings due to their novelty and frequently larger effect sizes [7] but basic research articles are also the most susceptible to sensationalisation [28]. While experienced scientists and many journalists likely know to view these papers as potentially useful pieces in a much greater puzzle, the general population may not have the experience or specialist knowledge to interpret individual reports critically in a broader context. Despite blame commonly being attributed to journalists and press releases, statements in the research articles themselves are often exaggerated and may generate hype [38, 39]. For example, many observational studies in high impact journals contain advice on clinical practice without mentioning the need for confirmation by RCTs [9] or fail to discuss population biases, small samples sizes, or difficulties in translating animal studies to humans.

The low occurrence of secondary studies reported in our sample of online reports is consistent with previous findings showing that systematic reviews represented a small proportion of medical research news [8]. This trend is likely reflected in the frequency of these studies published in journals, although we could not find any literature that directly investigated the publication frequency of different study types in medical research. We found a majority of online news articles reporting on peer-reviewed papers, however this may be partly explained by our exclusion of more general news articles that did not report on a specified study. Our analysis of reporting quality and study type distribution in online news is consistent with previous evidence of poor quality reporting by broadsheet news sources [12, 24, 26] and a bias towards primary research [14, 16, 40, 41]. This predominance of primary studies in the news increases the likelihood that the general public is left with a distorted perception of cancer research and an inaccurate view of scientific progress [2, 28, 38]. Reporting quality scores varied within and across the news sources. Mean quality scores indicated similarity between *The Guardian* and *NY Times* but were significantly lower in the Australian news sources. It should also be noted that availability of resources in different media outlets may have a significant influence on reporting quality but we were not able to measure this effect.

Previous analysis of cancer research stories on the *BBC* website from 1998–2006 found a heavy focus on breast cancer, followed by lung and prostate cancers [42]. Almost a quarter of news reports in our cohort did not refer to a specific cancer type, possibly reflecting the strong bias towards reporting of basic research on disease mechanisms and risk. However, an important underlying factor in this trend may also be the emergence of defining cancer type by molecular rather than anatomical classifiers [43, 44]. We observed a correlation between the representation of various cancer types (classified by anatomical site) and the relative incidence rates of those cancer types, but no relationship between reporting frequency and relative mortality rates. Cervical, melanoma and breast cancer were over-represented relative to their respective mortality rates, while lung, pancreas, and colorectal cancer were under-represented. Continued public and media interest on the effectiveness of the HPV vaccine *Gardasil*, and changes in screening practices may be a relevant consideration in the over-representation of reports focussing on cervical cancer. Over or under representation of different cancer types in research reporting may skew public awareness of risk factors and may also drive inequities in public and philanthropic funding of research directed as specific cancer types.

We observed a striking national bias, where news outlets were more likely to report on research performed in the same country they were based. Reporting research of relevance to distinct geographical areas (e.g. epidemiological investigations on specific populations) may be very important in informing the public with regards to local risk factors and outcomes of publicly funded research. Hence, the predominance of epidemiological studies in news reports is one possible contributor to the observed national bias. Conversely, prioritising reporting on local research means the public may not get access to important information from broader sources, although the modern online environment puts global news within reach of the majority of people.

The *Matilda effect*, which describes the systematic under-recognition of female scientists, has been extended to science communication, where greater perceived scientific quality is associated with publications from male authors [36, 37]. Further, under-representation of women in news media more generally is well established [34, 35]. We observed a striking gender bias in both study selection and reporting in our cohort, with the majority of reports based on studies with male senior authors and quoting male experts. This bias has potential to compromise high-quality coverage of research by limiting diversity of opinion, and likely serves to reinforcing stereotypes and further entrench gender inequity among researchers by providing public visibility and recognition predominantly to male scientists. The suite of biases faced by female scientists is well documented [30–33], and the gender bias in news reports likely reflects an underlying predominance of men among the ranks of senior scientists. The possibility of other underlying biases can’t be excluded, including differences in the availability and/or willingness of male experts to speak to journalists. Regardless, our data highlights a need for journalists, scientists and institutes to significantly improve efforts to ensure equal representation of male and female scientists in news reports on cancer research.

There are a number of assumptions and limitations to our study. As cancer research output is not expected to fluctuate notably throughout the year, it was assumed that a six-month sample period limited to 20 reports from each of the four news outlets would provide a representative cohort to analyse. An exception was conference-based news reports, which peaked at the time of a major cancer conference in June (ASCO annual meeting). In all news outlets apart from *The Guardian*, the 20 reports comprised the majority of relevant reports within the selected time frame and should thus be considered representative samples of each source. Although the size of our cohort limits a comprehensive representation of national reporting trends, the chosen sources are all major news outlets in their respective countries and so likely provide a reasonable indication of broader trends. It was also assumed that all studies could be classified according to the classifications used. Secondary studies were too rare to allow analysis of any potential relationship between study type and reporting quality, which should be investigated in larger datasets.

The quality indicators measured in our cohort provide useful guidelines for journalists to consider in providing the most informative and accurate reporting of research. These are particularly relevant to minimising the potential for hyperbole, providing an objective account of research outcomes and implications, and in assisting readers to critically assess the report. Our data highlight the importance of considering which research types are selected for coverage. Acknowledging the importance of journalists providing independent and novel context, interpretation and insight to individual stories, a better representation of the current state in cancer research would be achieved by attempting to cover a more balanced proportion of primary and secondary studies, from national and international sources. Pointing to areas for potential future improvement, reports in the Australian news outlets (SMH and ABC) often failed to consult an independent expert, provide a link to the research study, and avoid overgeneralisation. While all sources regularly failed to mention limitations of the study being discussed, Australian news more often omitted such statements. These limitations may reflect limited time and/or resources of reporters or a trend towards having fewer specialist reporters in Australian media, but we are unable to quantify these in our dataset.

Conversely, when communicating with the news media, scientists should be conscious of the possible discrepancy between impact in the scientific community and among the general public. Where possible, readers should be made aware of what type of study is being reported on, whether it is peer-reviewed and how strong the supporting evidence is. Both journalists and scientists should also take care to mention the limitations and caveats of novel ideas in research and be mindful of accurately conveying uncertainty.

As far as we know, this is the first combined analysis of study type distribution, reporting quality, and other biases in cancer research reporting. These data highlight the presence of significant biases and provide a basis for improving the selection of studies being selected for media coverage, and the way those studies are reported. Future analyses should build on the findings reported here by incorporating the long-term outcomes and impact of the studies that appear in the news media. It would also be useful to evaluate to what extent corrections follow in the news after one of these studies have been refuted or a declared ‘breakthrough drug’ fails to reach the market. Further, analyses of relationships between readership, study type and reporting quality would offer insight into how demand-driven these biases may be. Accurate, contextual reporting of cancer research is imperative in helping the public understand complex and challenging science and appreciate the outcomes of publicly funded research, avoid undermining trust in science, and assist informed decision-making.

## Supporting information

S1 Table Details of media reporting cohort, including source URL and quality scores. (XLSX)
